# Evidence against a role for platelet-derived molecules in liver regeneration after partial hepatectomy in humans

**Published:** 2016-08-03

**Authors:** Marc Kirschbaum, Jelle Adelmeijer, Edris M. Alkozai, Robert J. Porte, Ton Lisman

**Affiliations:** 1 *Surgical Research Laboratory, Department of Surgery, University of Groningen, University Medical Center Groningen, Groningen, the Netherlands*; 2 *Section of Hepatobiliairy Surgery and Liver Transplantation, Department of Surgery, University of Groningen, University Medical Center Groningen, Groningen, the Netherlands*

**Keywords:** platelet, liver resection, growth factors, liver regeneration, pylorus-preserving, pancreatico-duodenectomy

## Abstract

**Background and Aim:** Blood platelets have been shown to stimulate liver regeneration after partial hepatectomy in animal models and humans, but the molecular mechanisms involved are unclear. It has been proposed that growth factors and angiogenic molecules stored within platelets drive platelet-mediated liver regeneration, but little direct evidence in support of this mechanism is available.

**Methods:** We assessed levels of relevant platelet-derived proteins (vascular endothelial growth factor, hepatocyte growth factor, fibroblast growth factor, platelet-derived growth factor, thrombospondin, and endostatin) in platelet-rich and platelet-poor plasma taken at various perioperative time points from patients undergoing a (extended) right partial hepatectomy (*n* = 17) or a pylorus-preserving pancreatico-duodenectomy (*n* = 10). In addition, we collected intraoperative samples from the efferent and afferent liver veins prior to and after completion of liver resection. Twenty-four healthy controls were included to establish reference ranges for the various tests.

**Results and Conclusions:** Although we demonstrate perioperative changes in platelet and plasma levels of the proteins assessed, the changes observed in patients undergoing partial hepatectomy largely mirror the changes observed in patients undergoing a pylorus-preserving pancreatico-duodenectomy. In addition, no change in the growth factor levels in platelet-rich plasma between afferent and efferent liver veins was observed. Thus, the absence of an intra- or postoperative consumption of platelet-derived proteins in patients undergoing partial hepatectomy argues against a role of release of these molecules in stimulation of liver regeneration.

**Relevance for patients:** In depth knowledge of the mechanism underlying platelet-mediated liver regeneration may facilitate development of targeted therapeutic interventions for patients with failing liver regeneration, which for example may occur following a partial hepatectomy. Although the prevailing dogma is that platelet stimulate liver regeneration by release of growth factors stored within platelets, data in this manuscript argue against this mechanism and suggest other pathways to be responsible.

## 1. Introduction

Platelets are well known for their functions in thrombosis and hemostasis. In addition, platelets have roles beyond physiological or pathological thrombus formation [1-3]. Animal models and clinical studies have suggested a pivotal role for platelets in stimulation of liver regeneration [4-10]. Although there is broad consensus that platelets stimulate liver regeneration in animal models and likely also in humans, there is ongoing debate on the molecular mechanisms underlying platelet-mediated stimulation of liver regeneration [11,12]. Some studies suggest that local release of growth factors or angiogenic molecules stored within platelet alpha and dense granules are responsible for platelet-mediated liver regeneration [4,13-16]. Even though it has been reported that platelet-derived growth factors stimulate hepatocyte proliferation in vitro [13,14,17], it has not yet been established whether platelet-derived growth factors also drive platelet-mediated liver regeneration in vivo [12]. Animal studies and a single study in humans have demonstrated accumulation of platelets within the liver remnant rapidly following a partial liver resection [5,16,18]. Nevertheless, it has not been unequivocally demonstrated that platelet accumulation results in (selective) release of liver-directed growth factors, nor has it been demonstrated that platelet derived growth factors indeed drive platelet-mediated liver regeneration.

For example, animal studies have suggested that platelet-derived serotonin drive platelet-mediated liver regeneration [4,19]. Indeed, mice that lack serotonin within platelet dense granules have a clearly delayed regeneration [4]. However, as serotonin is a relevant platelet activator [20], the decreased functional capacity of serotonin deficient platelets, rather than a direct mitogenic effect of serotonin could explain the delay in liver regeneration in these mice. In addition, studies in humans have suggested consumption of platelet-derived serotonin [15] and release of other molecules (notably vascular endothelial growth factor) potentially driving platelet-mediated liver regeneration from platelets following a liver regeneration in humans [16]. Unfortunately, these studies did not control for effects of a major surgical procedure, and in our opinion these data thus not convincingly demonstrate a role for platelet-derived proteins in liver regeneration in humans [21].

Thus, even though there is consensus that platelets stimulate liver regeneration, the exact mechanisms involved have not been thoroughly studied. Besides release of growth factors from platelets, alternative scenarios that may underlie platelet-mediated liver regeneration have been postulated [11]. Such mechanisms include transfer of RNA from platelets to hepatocytes as we have recently described [18], and platelet-mediated influx of inflammatory cells to the regenerating liver [22]. The latter mechanism is yet unexplored in literature although it is well known that inflammatory cells drive liver regeneration and the interactions between platelets and inflammatory cells have been well established [23-25].

Here we studied the levels of multiple relevant platelet-derived growth factors and angiogenic proteins in patients undergoing a partial liver resection and compared the levels to patients undergoing a pylorus-preserving pancreatico-duodenectomy (PPPD) and healthy controls.

## 2. Material & Methods

### 2.1. Patients

Seventeen adult patients who underwent a right (segments 5-8, *n* = 15) or extended right (segments 4-9, *n* = 2) partial hepatectomy were included in this study. The control group consisted often patients who underwent a PPPD. Twenty-four adult healthy volunteers were included to establish reference values for the various tests performed. Details of patients and controls have been previously reported elsewhere [26,27]. In short, all patients were included in the University Medical Center Groningen, the Netherlands. Exclusion criteria were age younger than 18 years, pre-existing coagulation disorders, pre-operative anti-coagulation, and use of non-steroidal anti-inflammatory drugs or aspirin 1 week before surgery. Routine surgical and anesthesiological procedures were adopted. The study protocol was approved by the local medical ethical committee and informed consent was obtained from each subject before inclusion in the study.

### 2.2. Plasma samples

In both patient groups, plasma samples for analyses were drawn at the following time points: after induction of anesthesia (baseline), at the end of surgery, and on post-operative days 1, 3, 5, 7 and 30. In addition we collected intraoperative blood samples in the patients undergoing partial hepatectomy. We took blood samples from the portal vein and from the hepatic vein of the liver remnant just before the start and just after completion of anatomical transection. Following surgery, all patients received standard thromboprophylaxis with (once daily) low molecular weight heparin (LMWH) and at each post-operative day blood was drawn just prior to the administration of the LMWH. Blood samples from each subject were drawn by venipuncture and collected into vacuum tubes containing 3.8% trisodium citrate as an anti-coagulant, at a blood to anti-coagulant ratio of 9:1. Platelet counts in whole blood were determined by the diagnostic laboratory of our hospital. Platelet-rich plasma (PRP) was prepared by centrifugation at 250 g for 10 min. at room temperature. The platelet count in the PRP was determined by the diagnostic laboratory of our hospital and platelets were lysed by repeated free-thaw cycles and stored at –80°C until use. Platelet-poor plasma (PPP) was prepared by centrifugation of whole blood at 2000 g for 10 min at room temperature, with subsequent recentrifugation of the plasma fraction at 10,000 g for 10 min. at room temperature. Plasma was aliquoted, snap-frozen and stored at –80°C until use.

### 2.3. Assays for platelet-derived proteins

Levels of platelet-derived proteins were measured in PRP lysates and in PPP. Specifically, levels of vascular endothelial growth factor (VEGF), hepatocyte growth factor (HGF), fibroblast growth factor (FGF), platelet-derived growth factor (PDGF), thrombospondin 1 (TSP1), and endostatin were quantified using commercially available enzyme-linked immuno-sorbent assays (ELISAs). For all proteins DuoSet ELISA Development Systems (R&D Systems, Oxon, United Kingdom) were used according to the manufacturer’s instructions. Platelet residues in the PRP lysates were spun down prior to the assays. The concentration of growth factors within platelets was calculated by subtracting values obtained in PRP from those in corresponding PPP samples after which values were divided by the platelet count in the PRP sample.

### 2.4. Statistics

Values are expressed using box plots with medians, interquartile ranges, and minimal and maximal values indicated. Differences between values at a single time point between patients undergoing partial hepatectomy and patients undergoing a PPPD were assessed by the Mann-Whitney U-test. Differences between samples taken from the efferent and afferent liver veins were assessed using a paired t-test or the Wilcoxon matched pairs test as appropriate. Differences between patient values and levels measured in healthy controls were compared using one-way analysis of variance (with the Friedman test). *P* values of 0.05 or less were considered statistically significant.

## 3. Results

### 3.1. Patient characteristics

Seventeen patients who underwent a right (*n* = 15) or extended right (*n* = 2) partial hepatectomy, 10 patients who underwent a PPPD, and twenty-four controls were included in the study. The main characteristics of the study population have been reported elsewhere [26, 27]. The most common indication for liver resection was liver metastases from coloreetal cancer and the most common indication for PPPD was pancreatic cancer.

### 3.2. Platelet counts during and after partial hepatectomy or PPPD

**Figure 1.**
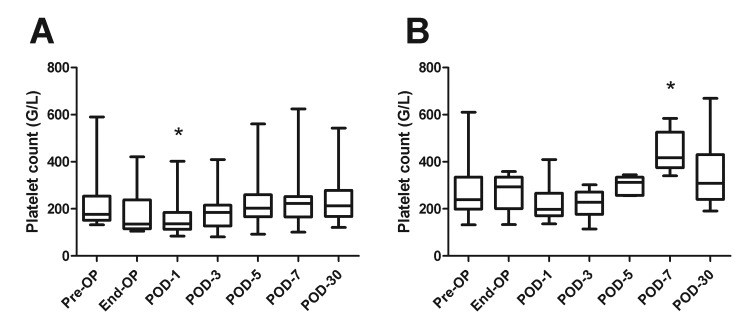
Circulating platelet count prior to and after partial liver resection or PPPD. *P < 0.05. Pre-OP, pre-operative, End-OP, end of surgery; POD, post-operative day.

Platelet counts were slightly lower at post-operative day 1 compared to baseline values in both patient groups (Figure 1). In the PPPD group, platelet counts were substantially elevated at post-operative day 7, while no clear increase in platelet count was observed in the partial hepatectomy group.

### 3.3. Levels of growth factors and angiogenic proteins in plateletrich plasma taken from the afferent and efferent liver veinsduring partial hepatectomy

We measured levels of various growth factors and angiogenic molecules in PRP taken from the afferent and efferent liver veins just before the start and just after completion of the parenchymal transection in patients undergoing a partial hepatectomy. As we were only able to draw a limited amount of blood from afferent and efferent veins, we only processed blood to PRP, and did not assess levels in PPP.

**Figure 2.**
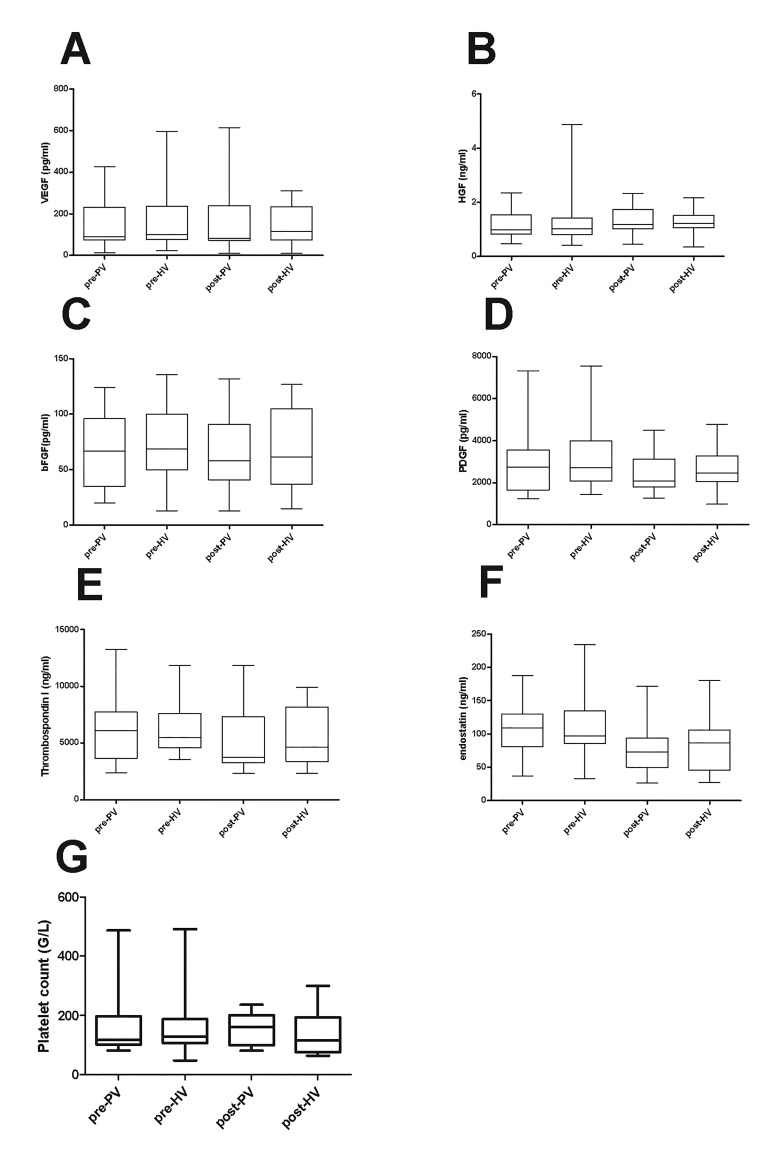
Levels of growth factors and angiogenic molecules in afferent and efferent veins prior to and just after anatomical transection in patients undergoing partial hepatectomy. Levels of (A) VEGF, (B) HGF, (C) bFGF, (D) PDGF, (E) TSP1, (F) endostatin, and (G) the platelet count in PRP were measured prior to (pre) and just after (post) parynchymal transection in samples taken from the portal vein (PV) or hepatic vein (HV) during partial hepatectomy.

Prior to and just after completion of the parenchymal transection levels of all proteins assessed were not different between afferent and efferent liver veins (Figure 2). Also, levels of counts measured in platelet rich plasma did not differ between all proteins were similar between both time points. Platelet the afferent and efferent liver veins both prior to and after completion of the parenchymal transection (*p* = 0.47 and *p* = 0.31, respectively, Figure 2G)

### 3.4. Levels of growth factors and angiogenic molecules inplasma and platelets taken at various perioperative time pointsfrom patients undergoing partial hepatectomy or PPPD

**Figure 3.**
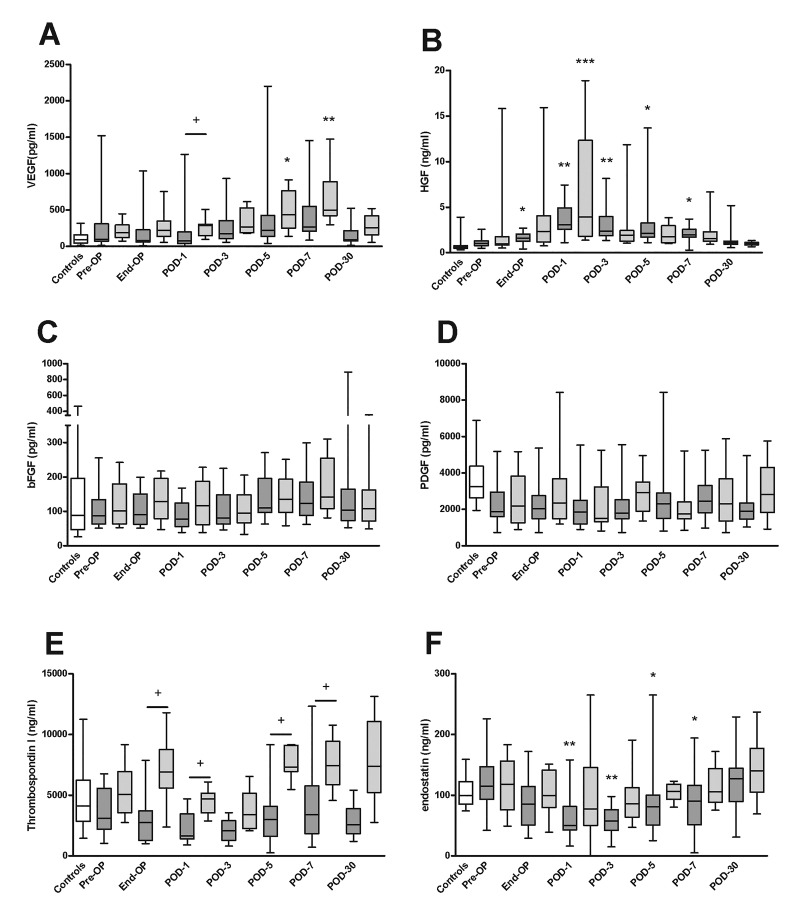
Levels of growth factors and angiogenic molecules in PRP taken at various perioperative time points from patients undergoing partial liver resection or PPPD. Levels of (A) VEGF, (B) HGF, (C) bFGF, (D) PDGF, (E) TSPl and (F) endostatin were measured in lysates of PRP in samples taken from healthy controls (white boxes), patients undergoing partial hepatectomy (dark grey boxes), and patients undergoing PPPD (light gray boxes). *P < 0.05, **P < 0.01, ***P < 0.001, versus pre-op (Friedman’s test). ^+^*P* < 0.05, partial hepatectomy versus PPPD (Mann-Whitney’s U test). Pre-OP, pre-operative, End-OP, end of surgery; POD, post-operative day.

**Figure 4.**
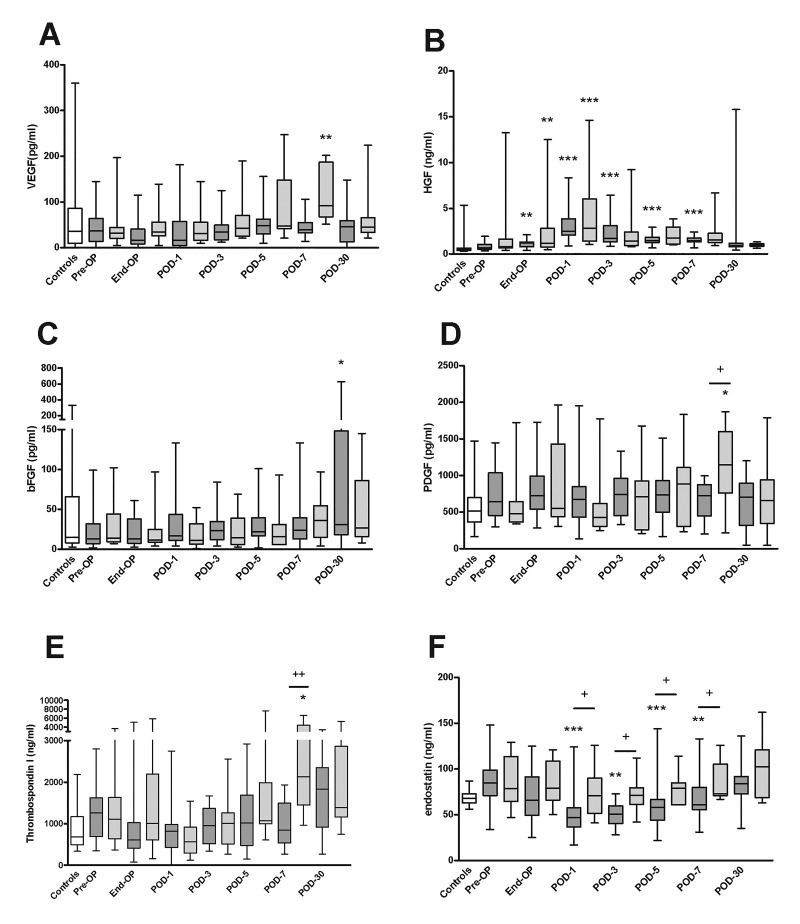
Levels of growth factors and angiogenic molecules in PPP taken at various perioperative time points from patients undergoing partial liver resection or PPPD. Plasma levels of (A) VEGF, (B) HGF, (C) bFGF, (D) PDGF, (E) TSPl and (F) endostatin in samples taken from healthy controls (white boxes), patients undergoing partial hepatectomy (dark grey boxes), and patients undergoing PPPD (light gray boxes).. *P < 0.05, **P < 0.01, ***p < 0.001, versus pre-op (Friedman’s test). ^+^*P* < 0.05, ^++^*P* < 0.01, hemihepatectomy versus PPPD (Mann-Whitney’s U test). Pre-OP, pre-operative, End-OP, end of surgery; POD, post-operative day.

**Figure 5.**
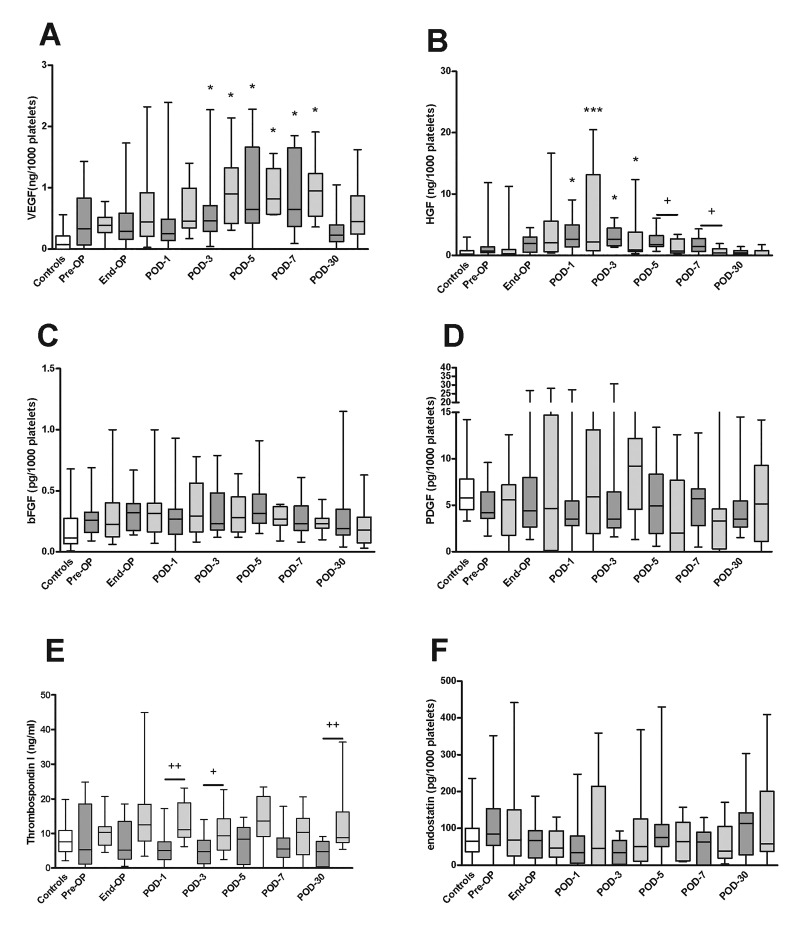
Levels of growth factors and angiogenic molecules in platelets taken at various perioperative time points from patients undergoing partial liver resection or PPPD. Calculated intraplatelet levels of (A) VEGF, (B) HGF, (C) bFGF, (D) PDGF, (E) TSPl and (F) endostatin in samples taken from healthy controls (white boxes), patients undergoing partial hepatectomy (dark grey boxes), and patients undergoing PPPD (light gray boxes).. *P < 0.05, **P < 0.01, ***p < 0.001, versus pre-op (Friedman’s test). ^+^*P* < 0.05, hemihepatectomy versus PPPD (Mann-Whitney’s U test). Pre-OP, pre-operative, End-OP, end of surgery; POD, post-operative day.

We measured levels of various growth factors and angiogenic proteins in PRP (Figure 3) and PPP (Figure 4), and calculated the amount of protein within platelets (Figure 5) in patients undergoing partial hepatectomy and PPPD and in healthy controls.

Levels of VEGF within platelets were slightly higher in both patient groups compared to controls, increased at post-operative day 3, and normalized again at post-operative day 30.

VEGF levels showed similar changes in the partial hepatectomy and PPPD groups. Plasma levels of VEGF did not appreciably change, except for an increase in the PPPD group at day 30.

HGF levels in plasma and in platelets increased from the end of surgery up to day 1, after which levels gradually decreased towards baseline. No differences between the partial hepatectomy and PPPD groups were detected, except for slightly higher levels within platelets in the partial hepatectomy group at days 5 and 7.

bFGF levels within platelets were slightly elevated compared to the control group at baseline, whereas plasma levels were not different between patients and controls. No changes in platelet or plasma bFGF levels occurred over time, and levels were not different between partial hepatectomy and PPPD patients.

PDGF levels in platelets and plasma did not significantly change over time, nor were levels different between the two patient groups.

Thrombospondin 1 levels within platelets were consistently higher in the PPPD group compared to the partial hepatectomy group, but no changes in platelet thrombospondin 1 levels occurred over time. Plasma levels of thrombospondin 1 did not change over time, and were similar between the PPPD and partial hepatectomy groups, except for an increase in the PPPD group on day 7.

Plasma levels of endostatin were lower in the patients undergoing partial hepatectomy between post-operative day 1 and 7, but levels within platelets were similar between the groups at all time points.

## 4. Discussion

In this study we found no difference between the circulating levels of six molecules that play important roles in liver regeneration between samples taken from the afferent and efferent liver veins immediately after a partial liver resection in humans. In addition, we found no clear differences in post-operative levels of these six proteins in platelets or plasma between patients that underwent a partial liver resection and patients that underwent a PPPD. Although levels of particularly VEGF and HGF changed over time, changes were similar between the partial hepatectomy and PPPD groups. Collectively, these results do not provide support for the hypothesis that selective release and consumption of proteins stored within platelets drives platelet-mediated liver regeneration.

Our results contradict recent clinical studies that have suggested consumption of serotonin and VEGF following a partial hepatectomy in humans [15,16]. Importantly, these studies did not control for the effects of major abdominal oncological surgery on levels of these molecules in platelets and/or plasma. Although it has been suggested that differences in sample processing may explain the divergent results obtained in our lab and the laboratory of Starlinger and coworkers [28,29], we feel there are other explanations for the divergent conclusions between our studies. First, the conclusion that consumption of VEGF and serotonin occurs following a partial hepatectomy in humans is not supported by our data in which we compared levels in PRP and PPP samples that have been processed in an identical fashion from patients undergoing partial liver resection and a PPPD. Although we did found changes in VEGF levels over time, these changes were very similar between the partial liver resection and PPPD groups. Similarly, we have recently reported that circulating serotonin levels similarly decrease in patients undergoing a partial liver resection and a PPPD [30]. Second, Starlinger and coworkers have reported accumulation of platelets in the liver remnant immediately after parenchymal transection which was accompanied by platelet activation and release of VEGF and thrombospondin [16]. In this and a previous study, we detected no changes in levels of serotonin [30] and the six proteins assayed in our present study between the afferent and efferent liver veins immediately following parenchymal transection, and found no evidence of a reduction in platelet count across the liver. We anticipate that surgical and anesthesiological differences, and differences in patient characteristics rather than differences in sample processing explain the apparent differences in secretion of platelet proteins within the liver between our studies.

It has been shown that VEGF and HGF plasma levels increase following a partial liver resection in humans [16,31], and these increases may directly contribute to liver regeneration. The regenerating liver synthesizes VEGF and HGF, presumably to aid its own regeneration [32,33]. It is therefore unclear why VEGF and HGF similarly increase in patients that underwent a PPPD. Our results, however, are in accordance with previously published data in which identical VEGF and HGF serum levels were shown at several time points following partial hepatectomy and pancreas resection [34]. These authors concluded that the increases in VEGF and HGF reflect wound healing and systemic inflammation as a result of surgical trauma. Similar conclusions were obtained in a study in which growth factor levels were compared between patients that underwent a partial liver resection and patients that underwent a laparotomy but did not undergo a partial hepatectomy as the tumor was found unresectable during surgery and in a study on VEGF levels following major abdominal surgery [35,36].

Baseline platelet VEGF, HGF, bFGF, and endostatin levels were higher in both patient groups compared to the controls, whereas plasma levels were very similar. These findings are in accordance with previous work that showed an increase in the levels of angiogenic proteins in platelets but not in plasma in both mouse models of cancer and in patients with cancer [37,38]. The consistently lower postoperative levels of the anti-angiogenic endostatin in patients undergoing partial hepatectomy as compared to the PPPD patients is of interest and may reflect the requirement for angiogenesis in the partial hepatectomy group.

Several limitations of our work must be acknowledged. First, the sample size of our study was small and variation in some of the analytes between patients was high. Consequently our conclusion that levels of the proteins assayed generally do not differ between patients undergoing a partial liver resection and a PPPD should be taken with caution. Secondly, it has been well established that plasma levels of platelet-derived proteins are very sensitive for pre-analytical variation. Although we had a standardized protocol to process blood, our protocol results in increased ex-vivo platelet activation compared to other published protocols [39]. Plasma levels of some of the analytes thus may be much higher than true ‘in vivo’ levels, although these falsely high values are likely present to a similar extent in all samples. Nevertheless, the extent of ex-vivo platelet activation does not lead to a substantial redistribution of proteins from platelet granules to plasma. For example, plasma thrombospondin levels are (according to published data and data obtained in our laboratory) ~10 fold higher using our blood processing protocol compared to the protocol recommended by Starlinger and coworkers [39]. However, the majority of thrombospondin is still within platelets (When comparing levels in PRP with that in PPP -12.5% of total thrombospondin is within platelets in our study, this level is -1.25% using our methodology in the Starlinger study [39], and -0.125% using the optimized methodology when comparing PPP versus serum levels as reported in that study). As we aimed to test the hypothesis that platelets (selectively) released proteins in patients undergoing liver (but not pancreas) resection, we feel that our final conclusion is justified despite these methodological issues. We do acknowledge that additional, larger studies with appropriate control groups are required to obtain definitive conclusions. Thirdly, one could argue that patients undergoing a PPPD are not ideal controls. We chose these patients as we then could compare two groups that both had major abdominal surgery for cancer with liver regeneration occurring in just one of the two groups. However, it has been suggested that the pancreas is also able to regenerate [40,41], and as platelets may potentially play a role in this process, comparison of liver- and pancreas resection may not be straightforward. On the other hand, there is no consensus in literature that pancreas regeneration occurs in humans [42,43]. Finally, we have only taken samples right after completion of parenchymal transection and at day 1. Possible consumption of analytes assessed in the first few hours after surgery, during which liver regeneration is already initiated, may therefore have been missed.

In conclusion, our data do not support the hypothesis that growth factors and angiogenic molecules in platelets are selectively released and consumed during liver regeneration after partial hepatectomy in humans. Although emerging evidence from clinical and experimental animal studies provides continuing support for the role of platelets in liver regeneration, the mechanism by which platelets stimulate this process may not involve a simple release of growth factors or angiogenic mediators from platelets.
